# Severe Cytomegalovirus Reactivation in Patient with Low-Grade Non-Hodgkin's Lymphoma after Standard Chemotherapy

**DOI:** 10.1155/2017/5762525

**Published:** 2017-10-22

**Authors:** Lena Modvig, Ciaran Boyle, Katie Randall, Anton Borg

**Affiliations:** Department of Haematology, Warwick Hospital, Warwick, UK

## Abstract

Clinically significant cytomegalovirus (CMV) reactivation is not uncommon in patients with severe immunodeficiency secondary to underlying medical disorders or following aggressive immunosuppressive therapy. However, it is less frequently found in patients with low-grade haematological malignancies after nonintensive chemotherapy. We treated a patient at our centre for stage IVB follicular lymphoma with standard chemotherapy who successively developed CMV colitis associated with a CMV viral load of >3 million copies/ml. Four lines of antiviral treatment were necessary to obtain biochemical remission with undetectable CMV levels, with an initially insufficient response to valganciclovir despite therapeutic pre- and posttreatment levels. Subsequently, our patient also developed an infection with *Pneumocystis jirovecii* pneumonia (PJP) as further evidence of severe immune compromise. This case report demonstrates the importance of including investigations for less common sources of infection when confronted with a patient with a low-grade haematological malignancy and a pyrexia of unknown origin.

## 1. Introduction

Cytomegalovirus (CMV) is a member of the Herpesvirus family that causes latent infection after the resolution of acute infection. Reactivation of CMV in the human host can arise at any time with the highest risk occurring with immunosuppression, either iatrogenic or secondary to systemic medical conditions [1–3]. Most commonly, CMV reactivation can be seen following solid organ transplants (SOTs) or transplantation of bone marrow stem cells which is known to cause severe immune compromise, as well as in critically ill patients [4–6]. Clinically significant CMV viraemia is much less frequently diagnosed in haematological patients with low-grade malignancies treated with nonintensive chemotherapy. We present a case of a 64-year-old male patient who underwent treatment at our centre for stage IVB follicular lymphoma (FL). Subsequently, he presented with CMV colitis and a CMV viral load of >3 million copies/ml. Four lines of antiviral treatment were necessary to obtain biochemical remission with undetectable CMV levels. Extensive immune impairment in our patient was made further evident when he later developed *Pneumocystis jirovecii* pneumonia (PJP).

## 2. Case Presentation

Our patient presented in 2011 with FL causing obstructive appendicitis on a background of extensive abdominal lymphadenopathy and was treated with eight cycles of rituximab, cyclophosphamide, vincristine, and prednisolone (R-CVP) to a good partial response. The chemotherapy was followed by rituximab maintenance, given every two months for two years. In 2015, a computerised tomography (CT) scan showed evidence of relapse with further investigations confirming reoccurrence of stage IVB FL. Second-line treatment with rituximab and bendamustine was commenced with a CT scan following three cycles of treatment showing a complete radiological response.

**Figure 1:**
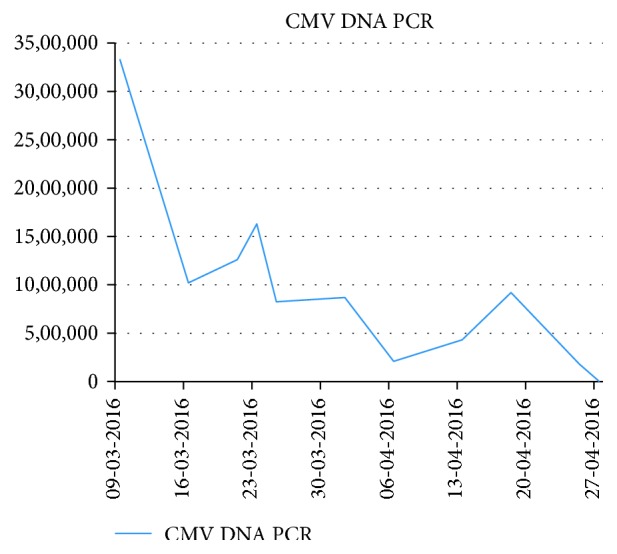
Levels of cytomegalovirus DNA by polymerase chain reaction recorded from time of identification of viral reactivation demonstrating a paraclinical response to antiviral medication.

Six days after cycle three of treatment, the patient was admitted to the hospital with lower abdominal pain, diarrhoea, and a pyrexia of unknown origin (PUO). Pericolonic inflammation of the hepatic flexure was detected on a CT scan of the abdomen. Extensive investigations including a full viral screen with polymerase chain reaction (PCR) for CMV showed a CMV viral load of 3,328,814 copies/ml (ref. <180 copies/ml) (Figure 1()), and treatment was commenced with ganciclovir 5 mg/kg for CMV colitis. After seven days, the medication was changed to cidofovir 5 mg/kg due to derangement of the liver function with a rise in ALT to 117 U/l (ref. 10–50 U/l), alkaline phosphatase 815 U/l (ref. 40–130 U/l), and gamma glutamyl transferase 1550 U/l (ref. 10–70 U/l). At this time, the CMV viral load had decreased to 812,307 copies/ml (Figure 1()). However, whilst on cidofovir, the CMV viral load increased to >1.6 million copies/ml, and weekly pulses of CMV immunoglobulin were added. This initially produced a response with a drop in the CMV viral load to 209,643 copies/ml followed by a rise to 920,330 copies/ml after a duration of twelve days. The treatment was changed to intravenous foscarnet given for two weeks, whereafter the patient was discharged on oral valganciclovir 450 mg twice daily (BD). At time of discharge, the CMV levels had dropped to 3405 copies/ml. Over the following six weeks, biweekly CMV levels as well as pre- and posttreatment valganciclovir levels to ensure therapeutic levels were performed. The dose of valganciclovir was increased to 900 mg BD upon which the CMV levels dropped to <180 copies/ml.

**Figure 2:**
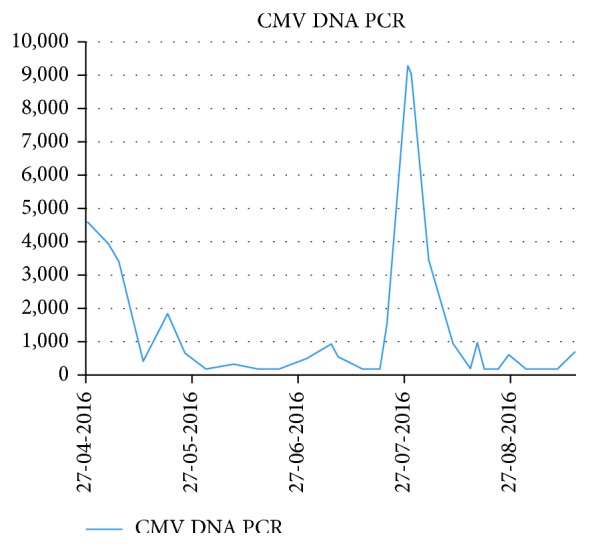
Levels of cytomegalovirus DNA by polymerase chain reaction recorded during patient's second hospital admission demonstrating a subsequent increase in viral load following an initially good response to treatment.

The patient was readmitted to the hospital with a PUO and CMV PCR levels remaining undetectable. Systematic investigations were performed including a bronchoscopy with alveolar lavage which was diagnostic of PJP. The patient was treated with high-dose trimethoprim and sulphamethoxazole (co-trimoxazole) given for three weeks and then discharged on a prophylactic dose of co-trimoxazole 480 mg daily. The CMV viral titre increased during the admission despite ongoing treatment with high-dose valganciclovir and remained detectable for two months following discharge (Figure 2()).

Dosage of valganciclovir had to be reduced to 450 mg BD due to persistent myelosuppression despite prophylactic injections with granulocyte colony-stimulating factor (G-CSF). With ongoing valganciclovir treatment and resolution of the PJP, CMV levels again became undetectable by PCR with a titre of <180 copies/ml on four successive blood tests. However, at the end of the study (14 September 2016), CMV levels had again started to increase (Figure 2()).

## 3. Discussion

We here present a case of severe and relatively treatment-resistant CMV reactivation in a patient with a low-grade non-Hodgkin's lymphoma (NHL). De novo viral infections as well as viral reactivations are not uncommon findings in patients undergoing treatment with bendamustine, with or without rituximab, for low-grade lymphoproliferative disorders. This is thought to be due to a variety of factors with influence on the immune system including lymphopenia with T- and B-cell depletion and hypogammaglobulinaemia [7]. Particularly, T-cell depletion associated with bendamustine is considered a major contributing factor to immune impairment against viral infections including CMV [8].

However, reactivation of CMV with elevation of viral titres to a level where it causes significant morbidity is not frequently found in patients with low-grade NHL treated with standard chemotherapy without stem cell transplantation. Previous studies have identified clinically significant CMV reactivation in only a minor subgroup of these patients [7, 9, 10], and to our knowledge, only a few cases have previously been reported [10–14]. This would indicate the infrequency of the phenomenon but also demonstrate the fact that CMV reactivation should be considered as a differential diagnosis in a patient with lymphoma and a PUO, regardless of the intensity of the chemotherapy regimen administered.

It is interesting to note that four lines of antiviral treatment were necessary to obtain undetectable levels of CMV by PCR. Initially, our patient did show a good response to first-line treatment with ganciclovir, but continued use of this drug was excluded due to adverse effects on the liver function. Also, the quite significant CMV viraemia >3 million copies/ml may have influenced the response to treatment, as previous studies have in fact demonstrated an association between low pretreatment CMV levels and faster time to disease resolution in SOT recipients [15].

Another interesting observation that has previously been found in patients with SOT is a lack of correlation between adequate ganciclovir plasma levels during treatment with valganciclovir and clearance of CMV [16]. In our case, pre- and posttreatment ganciclovir levels whilst on oral valganciclovir were performed on 3 occasions, and our patient was found to be within the therapeutic range despite showing an incomplete response to treatment by PCR of CMV DNA (data not shown).

Furthermore, an indication of considerable immunocompromise in our patient was made evident by the diagnosis of PJP when he presented with a PUO for the second time. In a manner similar to CMV reactivation, PJP most commonly presents in individuals with significantly suppressed immune systems, for example, as an AIDS-defining illness in patients with HIV or following aggressive immunosuppressive therapy [17–20].

This case report demonstrates the necessity of thinking outside the box and including investigations for less common sources of infection when confronted with a patient with a low-grade haematological malignancy and a PUO. We recommend clinicians have a low threshold for assessment of viral reactivation in this group of patients when the source of infection is not readily detectable. Identification of a possible scoring system to distinguish patients with low-grade NHL at risk of developing clinically significant CMV reactivation remains to be investigated in larger cohorts.
